# Tetra­kis(μ-3,4-dimethoxy­phenyl­acetato)bis­[(3,4-dimethoxy­phenyl­acetato)(1,10-phenanthroline)dysprosium(III)]

**DOI:** 10.1107/S1600536810005143

**Published:** 2010-02-13

**Authors:** Jian-Feng Liu, Xue-Dan Xu, Hua-Qiong Li, Guo-Liang Zhao

**Affiliations:** a Zhejiang Key Laboratory for Reactive Chemistry on Solid Surfaces, Institute of Physical Chemistry, Zhejiang Normal University, Jinhua, Zhejiang 321004, People’s Republic of China, and College of Chemistry and Life Science, Zhejiang Normal University, Jinhua 321004, Zhejiang, People’s Republic of China

## Abstract

The title centrosymmetric dinuclear dysprosium(III) complex, [Dy_2_(C_10_H_11_O_4_)_6_(C_12_H_8_N_2_)_2_] or [Dy(*L*)_3_phen]_2_, is comprised of six 3,4-dimethoxy­phenylacetate (*L*) anions, two 1,10-phenanthroline (phen) mol­ecules and two Dy^III^ ions. The Dy^III^ atom is nine-coordinated by seven O atoms from five *L* ligands and two N atoms from the phen mol­ecules. The *L* ligands are coordinated to the Dy^III^ ion in three coordination modes: chelating, bridging and bridging-tridentate. C—H⋯O hydrogen bonding interactions consolidate the crystal packing.

## Related literature

For related structures, see: Li *et al.* (2006 ▶, 2007 ▶). 
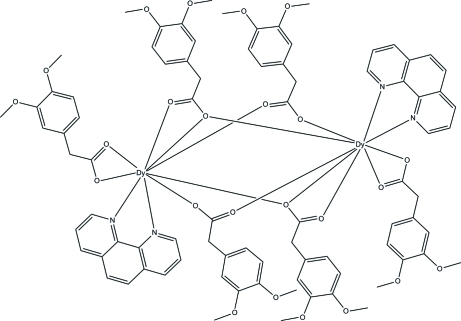


## Experimental

### 

#### Crystal data

[Dy_2_(C_10_H_11_O_4_)_6_(C_12_H_8_N_2_)_2_]
*M* *_r_* = 1856.54
Triclinic, 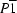

*a* = 12.3287 (2) Å
*b* = 12.3843 (3) Å
*c* = 14.6667 (3) Å
α = 90.968 (1)°
β = 103.461 (1)°
γ = 115.523 (1)°
*V* = 1947.70 (7) Å^3^
*Z* = 1
Mo *K*α radiation
μ = 1.99 mm^−1^
*T* = 296 K
0.43 × 0.19 × 0.07 mm

#### Data collection

Bruker APEXII CCD area-detector diffractometer
Absorption correction: multi-scan (*SADABS*; Sheldrick, 1996 ▶) *T*_min_ = 0.641, *T*_max_ = 0.874
26184 measured reflections
6861 independent reflections
5927 reflections with *I* > 2σ(*I*)
*R*_int_ = 0.035

#### Refinement

*R*[*F* ^2^ > 2σ(*F* ^2^)] = 0.028
*wR*(*F* ^2^) = 0.066
*S* = 1.03
6861 reflections
514 parameters
H-atom parameters constrained
Δρ_max_ = 0.62 e Å^−3^
Δρ_min_ = −0.42 e Å^−3^

### 

Data collection: *APEX2* (Bruker, 2006 ▶); cell refinement: *SAINT* (Bruker, 2006 ▶); data reduction: *SAINT*; program(s) used to solve structure: *SHELXS97* (Sheldrick, 2008 ▶); program(s) used to refine structure: *SHELXL97* (Sheldrick, 2008 ▶); molecular graphics: *SHELXTL* (Sheldrick, 2008 ▶); software used to prepare material for publication: *SHELXTL*.

## Supplementary Material

Crystal structure: contains datablocks I, global. DOI: 10.1107/S1600536810005143/pv2234sup1.cif

Structure factors: contains datablocks I. DOI: 10.1107/S1600536810005143/pv2234Isup2.hkl

Additional supplementary materials:  crystallographic information; 3D view; checkCIF report

## Figures and Tables

**Table 1:** Hydrogen-bond geometry (Å, °)

*D*—H⋯*A*	*D*—H	H⋯*A*	*D*⋯*A*	*D*—H⋯*A*
C1—H1*A*⋯O6^i^	0.96	2.54	3.320 (5)	138
C16—H16*A*⋯O3^ii^	0.93	2.53	3.425 (4)	161
C18—H18*B*⋯O3^ii^	0.96	2.36	3.262 (4)	156
C21—H21*A*⋯O1^iii^	0.96	2.49	3.362 (6)	151
C21—H21*A*⋯O2^iii^	0.96	2.43	3.214 (5)	139
C33—H33*A*⋯O7^iv^	0.93	2.38	3.231 (4)	153
C31—H31*A*⋯O4	0.93	2.52	2.977 (4)	111

Symmetry codes: (i) 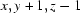; (ii) 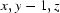; (iii) 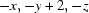; (iv) 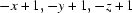.

**Fig. 1.**
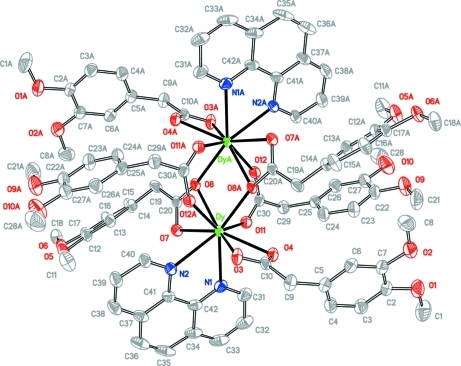
The molecular structure of the title complex. Displacement ellipsoids are drawn at the 30% probability level. The letter A in the atomic labels represents the symmetry operation: -x, -y+1, -z+1.

**Fig. 2.**
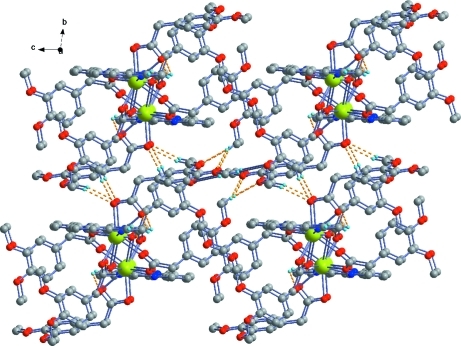
The packing plot of the title complex showing intermolecular interactions.

## supplementary crystallographic information

### Comment

The rare earth complexes with N/O-donors ligands such as 1,10-phenanthroline and carboxylic acid have received considerable attention for many years (Li *et al.*, 2007; Li *et al.*, 2006). The structure of the title compound, [Dy(*L*)_3_phen]_2_, (I), is a dinuclear dysprosium complex with Dy—DyA separation of 3.8887 (3) Å. The structure of the complex (Fig. 1) reveals that the molecule contains six 3,4-dimethoxyphenylacetic anions (*L*) and two 1,10-phenanthroline (phen) molecules and two Dy^III^ ions. The Dy^III^ ion is nine-coordinated by two N atoms from one phen and seven O atoms from (*L*) anions. The Dy—O bond distances range from 2.320 (2) to 2.522 (3) Å and the Dy—N bond distances are 2.536 (3) and 2.612 (3) Å, all of which are within the range of those of other nine-coordinated Dy^III^ complexes with carboxylic donor ligands and 1,10-phenanthroline (Li *et al.*, 2006). The (*L*) ligands are coordinated to the Dy^III^ ions in three different modes: chelating, bridging and bridging tridentate. Around each Dy^III^, there is one *L* ligand in chelating mode through two O atoms from the carboxyl group. Two symmetric *L* ligands bridge the two Dy centers though carboxyl O atoms. One *L* ligand is coordinated in a bidentate mode with Dy ion with O7 and O8 from carboxyl group and simultaneously bands to DyA ion with O8. In addition, there are no classical hydrogen bonds in the crystal structure, because good hydrogen bond donors are absent. The most significant intermolecular interactions are C—H···O hydrogen bonds (Table 1) and weak π···π aromatic interactions from phen molecules and aromatic rings of the *L* ligands. A packing plot is shown in Fig. 2.

### Experimental

A mixture of 3,4-dimethoxyphenylacetic acid (0.5886 g, 3 mmol), Dy_2_O_3_ (0.1865 g, 0.5 mmol), 1,10-phenanthroline (0.1982 g, 1 mmol) and purified water (20 ml) was sealed in a 25 ml stainless steel reactor and kept at 433 K for 3 d. Then, the reactor was cooled to room temperature at a speed of 5 degrees per hour. Lots of pink single crystals were filtered out of the mixture at high field (80%).

### Refinement

The H atoms bonded to C atoms were positioned geometrically and refined using a riding model with C—H distances: 0.96, and 0.93 Å for aliphatic and aromatic, respectively, and *U*_iso_(H) = 1.5*U*_eq_(*C*-methoxyl) or 1.2*U*_eq_(the rest of the parent atoms).

### Crystal data

**Table:**

[Dy_2_(C_10_H_11_O_4_)_6_(C_12_H_8_N_2_)_2_]	*Z* = 1
*M**_r_* = 1856.54	*F*(000) = 938
Triclinic, *P*1	*D*_x_ = 1.583 Mg m^−^^3^
Hall symbol: -p 1	Mo *K*α radiation, λ = 0.71073 Å
*a* = 12.3287 (2) Å	Cell parameters from 9399 reflections
*b* = 12.3843 (3) Å	θ = 1.8–25.0°
*c* = 14.6667 (3) Å	µ = 1.99 mm^−^^1^
α = 90.968 (1)°	*T* = 296 K
β = 103.461 (1)°	Plate, pink
γ = 115.523 (1)°	0.43 × 0.19 × 0.07 mm
*V* = 1947.70 (7) Å^3^	

### Data collection

**Table:**

Bruker APEXII CCD area-detector diffractometer	6861 independent reflections
Radiation source: fine-focus sealed tube	5927 reflections with *I* > 2σ(*I*)
graphite	*R*_int_ = 0.035
phi and ω scans	θ_max_ = 25.0°, θ_min_ = 1.8°
Absorption correction: multi-scan (*SADABS*; Sheldrick, 1996)	*h* = −13→14
*T*_min_ = 0.641, *T*_max_ = 0.874	*k* = −14→14
26184 measured reflections	*l* = −17→17

### Refinement

**Table:**

Refinement on *F*^2^	Primary atom site location: structure-invariant direct methods
Least-squares matrix: full	Secondary atom site location: difference Fourier map
*R*[*F*^2^ > 2σ(*F*^2^)] = 0.028	Hydrogen site location: inferred from neighbouring sites
*wR*(*F*^2^) = 0.066	H-atom parameters constrained
*S* = 1.03	*w* = 1/[σ^2^(*F*_o_^2^) + (0.0353*P*)^2^ + 0.1683*P*] where *P* = (*F*_o_^2^ + 2*F*_c_^2^)/3
6861 reflections	(Δ/σ)_max_ = 0.003
514 parameters	Δρ_max_ = 0.62 e Å^−^^3^
0 restraints	Δρ_min_ = −0.41 e Å^−^^3^

### Special details

**Table:**

Geometry. All e.s.d.'s (except the e.s.d. in the dihedral angle between two l.s. planes) are estimated using the full covariance matrix. The cell e.s.d.'s are taken into account individually in the estimation of e.s.d.'s in distances, angles and torsion angles; correlations between e.s.d.'s in cell parameters are only used when they are defined by crystal symmetry. An approximate (isotropic) treatment of cell e.s.d.'s is used for estimating e.s.d.'s involving l.s. planes.
Refinement. Refinement of *F*^2^ against ALL reflections. The weighted *R*-factor *wR* and goodness of fit *S* are based on *F*^2^, conventional *R*-factors *R* are based on *F*, with *F* set to zero for negative *F*^2^. The threshold expression of *F*^2^ > σ(*F*^2^) is used only for calculating *R*-factors(gt) *etc*. and is not relevant to the choice of reflections for refinement. *R*-factors based on *F*^2^ are statistically about twice as large as those based on *F*, and *R*- factors based on ALL data will be even larger.

### Fractional atomic coordinates and isotropic or equivalent isotropic displacement parameters (Å^2^)

**Table:**

	*x*	*y*	*z*	*U*_iso_*/*U*_eq_	
Dy	0.177981 (13)	0.606337 (14)	0.531660 (10)	0.04130 (7)	
N1	0.3869 (2)	0.6453 (2)	0.5047 (2)	0.0503 (7)	
O1	0.2349 (3)	1.0157 (3)	0.06745 (19)	0.0829 (9)	
C1	0.3116 (5)	1.0035 (5)	0.0144 (3)	0.1012 (17)	
H1A	0.2851	1.0170	−0.0494	0.152*	
H1B	0.3055	0.9235	0.0144	0.152*	
H1C	0.3965	1.0616	0.0422	0.152*	
N2	0.3613 (2)	0.6607 (2)	0.68308 (19)	0.0459 (7)	
C2	0.2595 (3)	0.9987 (3)	0.1610 (2)	0.0562 (9)	
O2	0.1032 (3)	1.0566 (3)	0.1614 (2)	0.0796 (8)	
O3	0.2631 (2)	0.8206 (2)	0.56316 (15)	0.0566 (6)	
C3	0.3471 (4)	0.9643 (4)	0.2054 (3)	0.0642 (10)	
H3A	0.3941	0.9475	0.1713	0.077*	
O4	0.2246 (2)	0.7496 (2)	0.41548 (15)	0.0475 (5)	
C4	0.3663 (4)	0.9542 (4)	0.3007 (3)	0.0642 (10)	
H4A	0.4269	0.9313	0.3305	0.077*	
O5	0.3890 (2)	0.2633 (2)	0.86802 (17)	0.0673 (7)	
C5	0.2964 (3)	0.9778 (3)	0.3524 (2)	0.0524 (9)	
O6	0.3680 (2)	0.0570 (2)	0.80505 (16)	0.0532 (6)	
C6	0.2069 (3)	1.0102 (3)	0.3066 (2)	0.0545 (9)	
H6A	0.1586	1.0251	0.3404	0.065*	
O7	0.2059 (2)	0.4291 (2)	0.58527 (16)	0.0494 (6)	
C7	0.1870 (3)	1.0212 (3)	0.2118 (3)	0.0566 (9)	
O8	0.01314 (19)	0.4012 (2)	0.54405 (14)	0.0460 (5)	
C8	0.0165 (4)	1.0668 (4)	0.2060 (4)	0.0875 (14)	
H8A	−0.0363	1.0931	0.1633	0.131*	
H8B	0.0609	1.1246	0.2621	0.131*	
H8C	−0.0335	0.9897	0.2226	0.131*	
O9	−0.1582 (3)	0.6792 (3)	−0.0064 (2)	0.0863 (9)	
C9	0.3152 (4)	0.9648 (3)	0.4557 (2)	0.0599 (10)	
H9A	0.2761	1.0058	0.4827	0.072*	
H9B	0.4039	1.0056	0.4865	0.072*	
C10	0.2645 (3)	0.8366 (3)	0.4783 (2)	0.0452 (8)	
O10	−0.2506 (3)	0.4530 (3)	−0.0641 (2)	0.0989 (11)	
O11	0.1139 (2)	0.4816 (2)	0.38781 (15)	0.0482 (6)	
C11	0.4211 (4)	0.3853 (4)	0.8958 (3)	0.0858 (14)	
H11A	0.4817	0.4130	0.9562	0.129*	
H11B	0.3479	0.3923	0.9005	0.129*	
H11C	0.4553	0.4335	0.8497	0.129*	
C12	0.3019 (3)	0.2086 (3)	0.7835 (2)	0.0449 (8)	
O12	−0.0940 (2)	0.3744 (2)	0.34464 (14)	0.0528 (6)	
C13	0.2243 (3)	0.2534 (3)	0.7331 (2)	0.0457 (8)	
H13A	0.2298	0.3261	0.7571	0.055*	
C14	0.1386 (3)	0.1928 (3)	0.6477 (2)	0.0464 (8)	
C15	0.1379 (3)	0.0886 (3)	0.6125 (2)	0.0589 (10)	
H15A	0.0858	0.0494	0.5531	0.071*	
C16	0.2124 (3)	0.0407 (3)	0.6630 (2)	0.0544 (9)	
H16A	0.2074	−0.0315	0.6383	0.065*	
C17	0.2931 (3)	0.0986 (3)	0.7490 (2)	0.0422 (7)	
C18	0.3356 (4)	−0.0674 (3)	0.7839 (3)	0.0643 (10)	
H18A	0.3933	−0.0875	0.8275	0.096*	
H18B	0.3389	−0.0830	0.7205	0.096*	
H18C	0.2527	−0.1157	0.7894	0.096*	
C19	0.0456 (3)	0.2354 (3)	0.5983 (3)	0.0555 (9)	
H19A	−0.0037	0.1815	0.5397	0.067*	
H19B	−0.0105	0.2259	0.6374	0.067*	
C20	0.0937 (3)	0.3622 (3)	0.5749 (2)	0.0405 (7)	
C21	−0.1059 (5)	0.8059 (4)	0.0225 (4)	0.1040 (18)	
H21A	−0.1436	0.8415	−0.0243	0.156*	
H21B	−0.0176	0.8419	0.0294	0.156*	
H21C	−0.1212	0.8195	0.0819	0.156*	
C22	−0.1141 (4)	0.6150 (4)	0.0525 (3)	0.0619 (10)	
C23	−0.0238 (4)	0.6649 (4)	0.1381 (3)	0.0674 (11)	
H23A	0.0099	0.7471	0.1584	0.081*	
C24	0.0166 (4)	0.5926 (4)	0.1936 (3)	0.0626 (10)	
H24A	0.0772	0.6267	0.2508	0.075*	
C25	−0.0327 (3)	0.4709 (3)	0.1643 (2)	0.0505 (9)	
C26	−0.1208 (3)	0.4213 (4)	0.0791 (3)	0.0609 (10)	
H26A	−0.1533	0.3393	0.0589	0.073*	
C27	−0.1623 (4)	0.4931 (4)	0.0224 (3)	0.0651 (10)	
C28	−0.2940 (5)	0.3346 (5)	−0.1046 (4)	0.1117 (19)	
H28A	−0.3547	0.3192	−0.1639	0.168*	
H28B	−0.3318	0.2802	−0.0628	0.168*	
H28C	−0.2257	0.3227	−0.1149	0.168*	
C29	0.0135 (3)	0.3945 (3)	0.2268 (2)	0.0553 (9)	
H29A	0.0978	0.4133	0.2250	0.066*	
H29B	−0.0391	0.3097	0.2030	0.066*	
C30	0.0115 (3)	0.4185 (3)	0.3286 (2)	0.0473 (8)	
C31	0.4017 (4)	0.6446 (3)	0.4185 (3)	0.0638 (10)	
H31A	0.3370	0.6388	0.3679	0.077*	
C32	0.5118 (4)	0.6525 (4)	0.4003 (3)	0.0741 (12)	
H32A	0.5205	0.6538	0.3390	0.089*	
C33	0.6049 (4)	0.6581 (3)	0.4738 (4)	0.0760 (13)	
H33A	0.6773	0.6611	0.4626	0.091*	
C34	0.5935 (3)	0.6595 (3)	0.5658 (3)	0.0620 (11)	
C35	0.6869 (3)	0.6620 (4)	0.6466 (4)	0.0800 (14)	
H35A	0.7584	0.6600	0.6378	0.096*	
C36	0.6733 (4)	0.6672 (4)	0.7337 (4)	0.0773 (13)	
H36A	0.7353	0.6686	0.7846	0.093*	
C37	0.5655 (3)	0.6707 (3)	0.7505 (3)	0.0567 (10)	
C38	0.5496 (4)	0.6802 (3)	0.8402 (3)	0.0664 (11)	
H38A	0.6120	0.6869	0.8931	0.080*	
C39	0.4428 (4)	0.6796 (3)	0.8510 (3)	0.0630 (10)	
H39A	0.4315	0.6863	0.9110	0.076*	
C40	0.3506 (3)	0.6686 (3)	0.7704 (2)	0.0537 (9)	
H40A	0.2772	0.6666	0.7785	0.064*	
C41	0.4684 (3)	0.6621 (3)	0.6729 (3)	0.0457 (8)	
C42	0.4820 (3)	0.6547 (3)	0.5790 (3)	0.0508 (9)	

### Atomic displacement parameters (Å^2^)

**Table:**

	*U*^11^	*U*^22^	*U*^33^	*U*^12^	*U*^13^	*U*^23^
Dy	0.04383 (10)	0.06268 (12)	0.04051 (10)	0.03999 (8)	0.01945 (7)	0.02237 (7)
N1	0.0516 (17)	0.0574 (18)	0.0621 (18)	0.0350 (14)	0.0295 (15)	0.0213 (14)
O1	0.094 (2)	0.120 (3)	0.0521 (16)	0.0582 (19)	0.0282 (15)	0.0339 (16)
C1	0.144 (5)	0.128 (4)	0.059 (3)	0.074 (4)	0.047 (3)	0.027 (3)
N2	0.0432 (15)	0.0532 (17)	0.0548 (17)	0.0325 (13)	0.0146 (13)	0.0193 (13)
C2	0.062 (2)	0.063 (2)	0.044 (2)	0.0268 (19)	0.0177 (18)	0.0211 (17)
O2	0.0745 (18)	0.105 (2)	0.081 (2)	0.0553 (17)	0.0263 (16)	0.0420 (17)
O3	0.0806 (17)	0.0676 (16)	0.0394 (13)	0.0464 (14)	0.0205 (12)	0.0201 (11)
C3	0.071 (3)	0.082 (3)	0.058 (2)	0.044 (2)	0.030 (2)	0.021 (2)
O4	0.0591 (14)	0.0579 (14)	0.0413 (12)	0.0367 (12)	0.0198 (11)	0.0157 (11)
C4	0.069 (2)	0.077 (3)	0.061 (2)	0.044 (2)	0.020 (2)	0.026 (2)
O5	0.0830 (18)	0.0541 (15)	0.0560 (15)	0.0393 (14)	−0.0142 (13)	0.0010 (12)
C5	0.061 (2)	0.047 (2)	0.051 (2)	0.0238 (17)	0.0186 (18)	0.0141 (16)
O6	0.0590 (14)	0.0567 (15)	0.0538 (14)	0.0407 (12)	0.0028 (11)	0.0115 (11)
C6	0.064 (2)	0.053 (2)	0.056 (2)	0.0290 (18)	0.0279 (18)	0.0168 (17)
O7	0.0439 (13)	0.0618 (15)	0.0610 (15)	0.0380 (12)	0.0175 (11)	0.0249 (12)
C7	0.057 (2)	0.055 (2)	0.062 (2)	0.0280 (18)	0.0192 (19)	0.0238 (18)
O8	0.0490 (13)	0.0713 (15)	0.0457 (13)	0.0476 (12)	0.0207 (10)	0.0274 (11)
C8	0.070 (3)	0.091 (3)	0.118 (4)	0.049 (3)	0.028 (3)	0.030 (3)
O9	0.102 (2)	0.089 (2)	0.0710 (19)	0.0555 (19)	0.0011 (16)	0.0217 (16)
C9	0.075 (3)	0.055 (2)	0.048 (2)	0.0282 (19)	0.0167 (19)	0.0128 (17)
C10	0.0458 (19)	0.064 (2)	0.0439 (19)	0.0392 (17)	0.0139 (15)	0.0185 (17)
O10	0.097 (2)	0.094 (2)	0.083 (2)	0.0435 (19)	−0.0172 (18)	−0.0099 (19)
O11	0.0475 (13)	0.0701 (16)	0.0469 (13)	0.0390 (12)	0.0223 (11)	0.0186 (12)
C11	0.104 (4)	0.055 (3)	0.082 (3)	0.042 (2)	−0.015 (3)	−0.009 (2)
C12	0.0491 (19)	0.0464 (19)	0.0419 (18)	0.0250 (16)	0.0090 (15)	0.0118 (15)
O12	0.0482 (13)	0.0853 (17)	0.0423 (13)	0.0424 (13)	0.0176 (11)	0.0165 (12)
C13	0.055 (2)	0.0414 (18)	0.051 (2)	0.0293 (16)	0.0150 (16)	0.0160 (15)
C14	0.0441 (18)	0.050 (2)	0.052 (2)	0.0282 (16)	0.0104 (16)	0.0193 (16)
C15	0.064 (2)	0.064 (2)	0.049 (2)	0.040 (2)	−0.0061 (17)	−0.0015 (18)
C16	0.064 (2)	0.060 (2)	0.050 (2)	0.0435 (19)	0.0037 (17)	0.0023 (17)
C17	0.0449 (18)	0.0499 (19)	0.0435 (18)	0.0303 (16)	0.0138 (15)	0.0157 (15)
C18	0.086 (3)	0.062 (2)	0.063 (2)	0.053 (2)	0.011 (2)	0.0158 (19)
C19	0.0443 (19)	0.057 (2)	0.070 (2)	0.0320 (17)	0.0051 (17)	0.0160 (18)
C20	0.0462 (19)	0.060 (2)	0.0358 (17)	0.0395 (17)	0.0145 (14)	0.0159 (15)
C21	0.128 (4)	0.084 (4)	0.100 (4)	0.055 (3)	0.013 (3)	0.043 (3)
C22	0.069 (2)	0.075 (3)	0.046 (2)	0.038 (2)	0.0133 (19)	0.018 (2)
C23	0.080 (3)	0.065 (3)	0.054 (2)	0.034 (2)	0.010 (2)	0.014 (2)
C24	0.069 (2)	0.070 (3)	0.047 (2)	0.033 (2)	0.0083 (18)	0.0149 (19)
C25	0.054 (2)	0.072 (3)	0.0380 (18)	0.0337 (19)	0.0226 (16)	0.0158 (17)
C26	0.063 (2)	0.069 (3)	0.055 (2)	0.032 (2)	0.0169 (19)	0.0088 (19)
C27	0.061 (2)	0.083 (3)	0.056 (2)	0.038 (2)	0.0115 (19)	0.016 (2)
C28	0.097 (4)	0.124 (5)	0.082 (4)	0.038 (3)	−0.007 (3)	−0.026 (3)
C29	0.064 (2)	0.077 (3)	0.048 (2)	0.047 (2)	0.0249 (17)	0.0145 (18)
C30	0.059 (2)	0.068 (2)	0.0456 (19)	0.0481 (19)	0.0263 (18)	0.0253 (17)
C31	0.068 (2)	0.076 (3)	0.074 (3)	0.045 (2)	0.041 (2)	0.025 (2)
C32	0.076 (3)	0.074 (3)	0.099 (3)	0.040 (2)	0.059 (3)	0.017 (2)
C33	0.051 (2)	0.057 (2)	0.137 (4)	0.028 (2)	0.050 (3)	0.007 (3)
C34	0.042 (2)	0.047 (2)	0.106 (3)	0.0225 (17)	0.033 (2)	0.007 (2)
C35	0.0288 (19)	0.060 (3)	0.149 (5)	0.0246 (18)	0.012 (2)	−0.007 (3)
C36	0.043 (2)	0.064 (3)	0.118 (4)	0.029 (2)	0.000 (2)	−0.004 (3)
C37	0.0387 (19)	0.042 (2)	0.085 (3)	0.0208 (16)	0.0018 (19)	0.0102 (19)
C38	0.057 (2)	0.054 (2)	0.076 (3)	0.0277 (19)	−0.010 (2)	0.015 (2)
C39	0.066 (2)	0.066 (3)	0.056 (2)	0.036 (2)	0.0031 (19)	0.0160 (19)
C40	0.055 (2)	0.064 (2)	0.054 (2)	0.0370 (18)	0.0116 (17)	0.0190 (18)
C41	0.0385 (17)	0.0358 (17)	0.067 (2)	0.0211 (14)	0.0123 (16)	0.0159 (16)
C42	0.0382 (18)	0.0408 (19)	0.085 (3)	0.0241 (15)	0.0231 (18)	0.0175 (18)

### Geometric parameters (Å, °)

**Table:**

Dy—O8^i^	2.320 (2)	C12—C17	1.395 (4)
Dy—O12^i^	2.343 (2)	O12—C30	1.257 (4)
Dy—O11	2.350 (2)	O12—Dy^i^	2.343 (2)
Dy—O3	2.385 (2)	C13—C14	1.385 (4)
Dy—O4	2.462 (2)	C13—H13A	0.9300
Dy—O7	2.478 (2)	C14—C15	1.377 (5)
Dy—O8	2.521 (2)	C14—C19	1.505 (4)
Dy—N1	2.536 (3)	C15—C16	1.383 (4)
Dy—N2	2.607 (3)	C15—H15A	0.9300
Dy—C10	2.780 (3)	C16—C17	1.366 (4)
Dy—C20	2.879 (3)	C16—H16A	0.9300
Dy—Dy^i^	3.8882 (3)	C18—H18A	0.9600
N1—C31	1.320 (4)	C18—H18B	0.9600
N1—C42	1.363 (4)	C18—H18C	0.9600
O1—C2	1.374 (4)	C19—C20	1.499 (5)
O1—C1	1.408 (5)	C19—H19A	0.9700
C1—H1A	0.9600	C19—H19B	0.9700
C1—H1B	0.9600	C21—H21A	0.9600
C1—H1C	0.9600	C21—H21B	0.9600
N2—C40	1.324 (4)	C21—H21C	0.9600
N2—C41	1.355 (4)	C22—C27	1.382 (5)
C2—C3	1.360 (5)	C22—C23	1.388 (5)
C2—C7	1.397 (5)	C23—C24	1.390 (5)
O2—C7	1.361 (4)	C23—H23A	0.9300
O2—C8	1.423 (5)	C24—C25	1.375 (5)
O3—C10	1.267 (4)	C24—H24A	0.9300
C3—C4	1.379 (5)	C25—C26	1.374 (5)
C3—H3A	0.9300	C25—C29	1.519 (5)
O4—C10	1.245 (4)	C26—C27	1.401 (5)
C4—C5	1.385 (5)	C26—H26A	0.9300
C4—H4A	0.9300	C28—H28A	0.9600
O5—C12	1.369 (4)	C28—H28B	0.9600
O5—C11	1.413 (4)	C28—H28C	0.9600
C5—C6	1.369 (5)	C29—C30	1.525 (4)
C5—C9	1.500 (5)	C29—H29A	0.9700
O6—C17	1.366 (4)	C29—H29B	0.9700
O6—C18	1.423 (4)	C31—C32	1.406 (5)
C6—C7	1.374 (5)	C31—H31A	0.9300
C6—H6A	0.9300	C32—C33	1.355 (6)
O7—C20	1.238 (4)	C32—H32A	0.9300
O8—C20	1.279 (3)	C33—C34	1.389 (6)
O8—Dy^i^	2.320 (2)	C33—H33A	0.9300
C8—H8A	0.9600	C34—C42	1.408 (4)
C8—H8B	0.9600	C34—C35	1.436 (6)
C8—H8C	0.9600	C35—C36	1.331 (6)
O9—C22	1.360 (4)	C35—H35A	0.9300
O9—C21	1.427 (5)	C36—C37	1.424 (5)
C9—C10	1.509 (5)	C36—H36A	0.9300
C9—H9A	0.9700	C37—C38	1.385 (5)
C9—H9B	0.9700	C37—C41	1.411 (5)
O10—C27	1.383 (5)	C38—C39	1.359 (5)
O10—C28	1.391 (6)	C38—H38A	0.9300
O11—C30	1.255 (4)	C39—C40	1.394 (5)
C11—H11A	0.9600	C39—H39A	0.9300
C11—H11B	0.9600	C40—H40A	0.9300
C11—H11C	0.9600	C41—C42	1.432 (5)
C12—C13	1.383 (4)		
			
O8^i^—Dy—O12^i^	75.76 (7)	O3—C10—Dy	58.83 (17)
O8^i^—Dy—O11	75.61 (8)	C9—C10—Dy	176.4 (2)
O12^i^—Dy—O11	138.17 (8)	C27—O10—C28	118.5 (4)
O8^i^—Dy—O3	89.20 (8)	C30—O11—Dy	135.4 (2)
O12^i^—Dy—O3	79.24 (8)	O5—C11—H11A	109.5
O11—Dy—O3	129.88 (7)	O5—C11—H11B	109.5
O8^i^—Dy—O4	75.39 (7)	H11A—C11—H11B	109.5
O12^i^—Dy—O4	124.07 (8)	O5—C11—H11C	109.5
O11—Dy—O4	76.27 (7)	H11A—C11—H11C	109.5
O3—Dy—O4	53.63 (7)	H11B—C11—H11C	109.5
O8^i^—Dy—O7	124.00 (7)	O5—C12—C13	125.1 (3)
O12^i^—Dy—O7	93.54 (8)	O5—C12—C17	115.2 (3)
O11—Dy—O7	78.13 (8)	C13—C12—C17	119.7 (3)
O3—Dy—O7	143.46 (8)	C30—O12—Dy^i^	137.0 (2)
O4—Dy—O7	142.07 (7)	C12—C13—C14	121.6 (3)
O8^i^—Dy—O8	73.21 (8)	C12—C13—H13A	119.2
O12^i^—Dy—O8	71.42 (8)	C14—C13—H13A	119.2
O11—Dy—O8	71.51 (7)	C15—C14—C13	117.2 (3)
O3—Dy—O8	148.60 (8)	C15—C14—C19	121.4 (3)
O4—Dy—O8	139.39 (7)	C13—C14—C19	121.3 (3)
O7—Dy—O8	51.68 (7)	C14—C15—C16	121.9 (3)
O8^i^—Dy—N1	141.81 (8)	C14—C15—H15A	119.1
O12^i^—Dy—N1	139.40 (9)	C16—C15—H15A	119.1
O11—Dy—N1	79.07 (8)	C17—C16—C15	120.4 (3)
O3—Dy—N1	85.52 (9)	C17—C16—H16A	119.8
O4—Dy—N1	71.12 (8)	C15—C16—H16A	119.8
O7—Dy—N1	76.92 (8)	C16—C17—O6	124.6 (3)
O8—Dy—N1	124.31 (8)	C16—C17—C12	118.9 (3)
O8^i^—Dy—N2	150.45 (8)	O6—C17—C12	116.4 (3)
O12^i^—Dy—N2	76.08 (8)	O6—C18—H18A	109.5
O11—Dy—N2	133.00 (8)	O6—C18—H18B	109.5
O3—Dy—N2	76.91 (8)	H18A—C18—H18B	109.5
O4—Dy—N2	114.21 (8)	O6—C18—H18C	109.5
O7—Dy—N2	66.60 (8)	H18A—C18—H18C	109.5
O8—Dy—N2	105.80 (7)	H18B—C18—H18C	109.5
N1—Dy—N2	63.83 (9)	C20—C19—C14	118.3 (3)
O8^i^—Dy—C10	81.72 (8)	C20—C19—H19A	107.7
O12^i^—Dy—C10	102.46 (9)	C14—C19—H19A	107.7
O11—Dy—C10	102.86 (9)	C20—C19—H19B	107.7
O3—Dy—C10	27.04 (8)	C14—C19—H19B	107.7
O4—Dy—C10	26.60 (8)	H19A—C19—H19B	107.1
O7—Dy—C10	152.75 (8)	O7—C20—O8	119.9 (3)
O8—Dy—C10	154.93 (8)	O7—C20—C19	123.0 (3)
N1—Dy—C10	76.57 (9)	O8—C20—C19	117.1 (3)
N2—Dy—C10	95.80 (9)	O7—C20—Dy	58.91 (17)
O8^i^—Dy—C20	99.15 (8)	O8—C20—Dy	61.01 (16)
O12^i^—Dy—C20	81.93 (8)	C19—C20—Dy	177.8 (2)
O11—Dy—C20	73.28 (8)	O9—C21—H21A	109.5
O3—Dy—C20	156.83 (8)	O9—C21—H21B	109.5
O4—Dy—C20	149.44 (8)	H21A—C21—H21B	109.5
O7—Dy—C20	25.34 (7)	O9—C21—H21C	109.5
O8—Dy—C20	26.35 (7)	H21A—C21—H21C	109.5
N1—Dy—C20	100.38 (9)	H21B—C21—H21C	109.5
N2—Dy—C20	85.57 (8)	O9—C22—C27	116.4 (3)
C10—Dy—C20	175.59 (9)	O9—C22—C23	124.1 (4)
O8^i^—Dy—Dy^i^	38.37 (5)	C27—C22—C23	119.4 (4)
O12^i^—Dy—Dy^i^	69.31 (5)	C22—C23—C24	120.3 (4)
O11—Dy—Dy^i^	69.27 (5)	C22—C23—H23A	119.9
O3—Dy—Dy^i^	123.04 (6)	C24—C23—H23A	119.9
O4—Dy—Dy^i^	109.98 (5)	C25—C24—C23	120.4 (3)
O7—Dy—Dy^i^	86.08 (5)	C25—C24—H24A	119.8
O8—Dy—Dy^i^	34.84 (5)	C23—C24—H24A	119.8
N1—Dy—Dy^i^	146.56 (7)	C26—C25—C24	119.6 (4)
N2—Dy—Dy^i^	134.08 (6)	C26—C25—C29	121.4 (3)
C10—Dy—Dy^i^	120.10 (6)	C24—C25—C29	119.0 (3)
C20—Dy—Dy^i^	60.91 (6)	C25—C26—C27	120.6 (4)
C31—N1—C42	118.8 (3)	C25—C26—H26A	119.7
C31—N1—Dy	121.0 (2)	C27—C26—H26A	119.7
C42—N1—Dy	119.9 (2)	C22—C27—O10	114.7 (4)
C2—O1—C1	117.3 (3)	C22—C27—C26	119.7 (4)
O1—C1—H1A	109.5	O10—C27—C26	125.6 (4)
O1—C1—H1B	109.5	O10—C28—H28A	109.5
H1A—C1—H1B	109.5	O10—C28—H28B	109.5
O1—C1—H1C	109.5	H28A—C28—H28B	109.5
H1A—C1—H1C	109.5	O10—C28—H28C	109.5
H1B—C1—H1C	109.5	H28A—C28—H28C	109.5
C40—N2—C41	117.1 (3)	H28B—C28—H28C	109.5
C40—N2—Dy	124.0 (2)	C25—C29—C30	110.7 (3)
C41—N2—Dy	118.0 (2)	C25—C29—H29A	109.5
C3—C2—O1	125.5 (4)	C30—C29—H29A	109.5
C3—C2—C7	119.7 (3)	C25—C29—H29B	109.5
O1—C2—C7	114.8 (3)	C30—C29—H29B	109.5
C7—O2—C8	117.9 (3)	H29A—C29—H29B	108.1
C10—O3—Dy	94.1 (2)	O11—C30—O12	126.1 (3)
C2—C3—C4	120.2 (4)	O11—C30—C29	117.6 (3)
C2—C3—H3A	119.9	O12—C30—C29	116.2 (3)
C4—C3—H3A	119.9	N1—C31—C32	122.4 (4)
C10—O4—Dy	91.09 (19)	N1—C31—H31A	118.8
C3—C4—C5	120.8 (4)	C32—C31—H31A	118.8
C3—C4—H4A	119.6	C33—C32—C31	118.8 (4)
C5—C4—H4A	119.6	C33—C32—H32A	120.6
C12—O5—C11	117.3 (3)	C31—C32—H32A	120.6
C6—C5—C4	118.4 (3)	C32—C33—C34	120.7 (4)
C6—C5—C9	120.5 (3)	C32—C33—H33A	119.6
C4—C5—C9	121.1 (4)	C34—C33—H33A	119.6
C17—O6—C18	116.2 (3)	C33—C34—C42	117.3 (4)
C5—C6—C7	121.5 (3)	C33—C34—C35	123.6 (4)
C5—C6—H6A	119.2	C42—C34—C35	119.0 (4)
C7—C6—H6A	119.2	C36—C35—C34	121.3 (4)
C20—O7—Dy	95.75 (19)	C36—C35—H35A	119.4
O2—C7—C6	125.4 (4)	C34—C35—H35A	119.4
O2—C7—C2	115.2 (3)	C35—C36—C37	121.3 (4)
C6—C7—C2	119.3 (4)	C35—C36—H36A	119.3
C20—O8—Dy^i^	158.0 (2)	C37—C36—H36A	119.3
C20—O8—Dy	92.64 (19)	C38—C37—C41	117.5 (3)
Dy^i^—O8—Dy	106.79 (8)	C38—C37—C36	123.1 (4)
O2—C8—H8A	109.5	C41—C37—C36	119.4 (4)
O2—C8—H8B	109.5	C39—C38—C37	120.1 (3)
H8A—C8—H8B	109.5	C39—C38—H38A	120.0
O2—C8—H8C	109.5	C37—C38—H38A	120.0
H8A—C8—H8C	109.5	C38—C39—C40	118.7 (4)
H8B—C8—H8C	109.5	C38—C39—H39A	120.7
C22—O9—C21	116.9 (3)	C40—C39—H39A	120.7
C5—C9—C10	115.4 (3)	N2—C40—C39	123.9 (4)
C5—C9—H9A	108.4	N2—C40—H40A	118.0
C10—C9—H9A	108.4	C39—C40—H40A	118.0
C5—C9—H9B	108.4	N2—C41—C37	122.8 (3)
C10—C9—H9B	108.4	N2—C41—C42	117.8 (3)
H9A—C9—H9B	107.5	C37—C41—C42	119.4 (3)
O4—C10—O3	121.1 (3)	N1—C42—C34	121.9 (4)
O4—C10—C9	121.1 (3)	N1—C42—C41	118.7 (3)
O3—C10—C9	117.8 (3)	C34—C42—C41	119.4 (3)
O4—C10—Dy	62.31 (17)		
			
O8^i^—Dy—N1—C31	−15.6 (3)	O8^i^—Dy—C10—O3	104.72 (19)
O12^i^—Dy—N1—C31	−166.3 (2)	O12^i^—Dy—C10—O3	31.4 (2)
O11—Dy—N1—C31	33.5 (3)	O11—Dy—C10—O3	177.81 (18)
O3—Dy—N1—C31	−98.5 (3)	O4—Dy—C10—O3	178.5 (3)
O4—Dy—N1—C31	−45.5 (3)	O7—Dy—C10—O3	−93.2 (3)
O7—Dy—N1—C31	113.7 (3)	O8—Dy—C10—O3	104.1 (2)
O8—Dy—N1—C31	92.0 (3)	N1—Dy—C10—O3	−106.9 (2)
N2—Dy—N1—C31	−176.1 (3)	N2—Dy—C10—O3	−45.61 (19)
C10—Dy—N1—C31	−72.7 (3)	Dy^i^—Dy—C10—O3	104.51 (18)
C20—Dy—N1—C31	104.1 (3)	O8^i^—Dy—O11—C30	−22.2 (3)
Dy^i^—Dy—N1—C31	52.4 (3)	O12^i^—Dy—O11—C30	25.9 (3)
O8^i^—Dy—N1—C42	171.3 (2)	O3—Dy—O11—C30	−98.7 (3)
O12^i^—Dy—N1—C42	20.6 (3)	O4—Dy—O11—C30	−100.3 (3)
O11—Dy—N1—C42	−139.6 (2)	O7—Dy—O11—C30	107.9 (3)
O3—Dy—N1—C42	88.3 (2)	O8—Dy—O11—C30	54.6 (3)
O4—Dy—N1—C42	141.3 (3)	N1—Dy—O11—C30	−173.3 (3)
O7—Dy—N1—C42	−59.4 (2)	N2—Dy—O11—C30	149.2 (3)
O8—Dy—N1—C42	−81.1 (3)	C10—Dy—O11—C30	−100.0 (3)
N2—Dy—N1—C42	10.7 (2)	C20—Dy—O11—C30	82.2 (3)
C10—Dy—N1—C42	114.2 (3)	Dy^i^—Dy—O11—C30	17.6 (3)
C20—Dy—N1—C42	−69.1 (2)	C11—O5—C12—C13	−14.3 (5)
Dy^i^—Dy—N1—C42	−120.8 (2)	C11—O5—C12—C17	166.5 (3)
O8^i^—Dy—N2—C40	24.7 (3)	O5—C12—C13—C14	179.8 (3)
O12^i^—Dy—N2—C40	6.7 (3)	C17—C12—C13—C14	−1.0 (5)
O11—Dy—N2—C40	−138.2 (2)	C12—C13—C14—C15	−3.1 (5)
O3—Dy—N2—C40	88.7 (3)	C12—C13—C14—C19	173.6 (3)
O4—Dy—N2—C40	128.1 (3)	C13—C14—C15—C16	4.9 (5)
O7—Dy—N2—C40	−93.5 (3)	C19—C14—C15—C16	−171.9 (3)
O8—Dy—N2—C40	−59.0 (3)	C14—C15—C16—C17	−2.4 (6)
N1—Dy—N2—C40	−179.9 (3)	C15—C16—C17—O6	178.3 (3)
C10—Dy—N2—C40	108.1 (3)	C15—C16—C17—C12	−1.9 (5)
C20—Dy—N2—C40	−76.1 (3)	C18—O6—C17—C16	−17.1 (5)
Dy^i^—Dy—N2—C40	−35.0 (3)	C18—O6—C17—C12	163.1 (3)
O8^i^—Dy—N2—C41	−166.39 (19)	O5—C12—C17—C16	−177.2 (3)
O12^i^—Dy—N2—C41	175.6 (2)	C13—C12—C17—C16	3.6 (5)
O11—Dy—N2—C41	30.7 (3)	O5—C12—C17—O6	2.7 (4)
O3—Dy—N2—C41	−102.4 (2)	C13—C12—C17—O6	−176.6 (3)
O4—Dy—N2—C41	−63.0 (2)	C15—C14—C19—C20	−127.0 (4)
O7—Dy—N2—C41	75.4 (2)	C13—C14—C19—C20	56.4 (5)
O8—Dy—N2—C41	109.9 (2)	Dy—O7—C20—O8	0.4 (3)
N1—Dy—N2—C41	−11.0 (2)	Dy—O7—C20—C19	−178.8 (3)
C10—Dy—N2—C41	−83.0 (2)	Dy^i^—O8—C20—O7	152.0 (4)
C20—Dy—N2—C41	92.8 (2)	Dy—O8—C20—O7	−0.4 (3)
Dy^i^—Dy—N2—C41	133.93 (19)	Dy^i^—O8—C20—C19	−28.7 (7)
C1—O1—C2—C3	−4.0 (6)	Dy—O8—C20—C19	178.8 (3)
C1—O1—C2—C7	175.2 (4)	Dy^i^—O8—C20—Dy	152.4 (5)
O8^i^—Dy—O3—C10	−73.18 (19)	C14—C19—C20—O7	7.9 (5)
O12^i^—Dy—O3—C10	−148.8 (2)	C14—C19—C20—O8	−171.3 (3)
O11—Dy—O3—C10	−2.8 (2)	O8^i^—Dy—C20—O7	169.48 (18)
O4—Dy—O3—C10	−0.82 (17)	O12^i^—Dy—C20—O7	−116.45 (19)
O7—Dy—O3—C10	129.84 (19)	O11—Dy—C20—O7	97.62 (19)
O8—Dy—O3—C10	−127.92 (19)	O3—Dy—C20—O7	−80.6 (3)
N1—Dy—O3—C10	68.97 (19)	O4—Dy—C20—O7	92.8 (2)
N2—Dy—O3—C10	133.1 (2)	O8—Dy—C20—O7	179.6 (3)
C20—Dy—O3—C10	175.0 (2)	N1—Dy—C20—O7	22.5 (2)
Dy^i^—Dy—O3—C10	−92.39 (18)	N2—Dy—C20—O7	−39.90 (19)
O1—C2—C3—C4	177.7 (4)	Dy^i^—Dy—C20—O7	172.8 (2)
C7—C2—C3—C4	−1.5 (6)	O8^i^—Dy—C20—O8	−10.1 (2)
O8^i^—Dy—O4—C10	100.86 (19)	O12^i^—Dy—C20—O8	63.97 (17)
O12^i^—Dy—O4—C10	39.8 (2)	O11—Dy—C20—O8	−81.97 (17)
O11—Dy—O4—C10	179.28 (19)	O3—Dy—C20—O8	99.8 (3)
O3—Dy—O4—C10	0.83 (17)	O4—Dy—C20—O8	−86.8 (2)
O7—Dy—O4—C10	−131.87 (19)	O7—Dy—C20—O8	−179.6 (3)
O8—Dy—O4—C10	141.16 (18)	N1—Dy—C20—O8	−157.13 (17)
N1—Dy—O4—C10	−97.76 (19)	N2—Dy—C20—O8	140.51 (17)
N2—Dy—O4—C10	−49.4 (2)	Dy^i^—Dy—C20—O8	−6.79 (14)
C20—Dy—O4—C10	−175.94 (18)	C21—O9—C22—C27	−177.9 (4)
Dy^i^—Dy—O4—C10	117.75 (17)	C21—O9—C22—C23	0.8 (6)
C2—C3—C4—C5	0.6 (6)	O9—C22—C23—C24	−179.5 (4)
C3—C4—C5—C6	0.7 (6)	C27—C22—C23—C24	−0.8 (6)
C3—C4—C5—C9	178.8 (3)	C22—C23—C24—C25	−0.1 (6)
C4—C5—C6—C7	−1.0 (5)	C23—C24—C25—C26	1.0 (5)
C9—C5—C6—C7	−179.1 (3)	C23—C24—C25—C29	−179.9 (3)
O8^i^—Dy—O7—C20	−12.6 (2)	C24—C25—C26—C27	−1.0 (5)
O12^i^—Dy—O7—C20	62.65 (19)	C29—C25—C26—C27	180.0 (3)
O11—Dy—O7—C20	−75.93 (19)	O9—C22—C27—O10	−0.5 (5)
O3—Dy—O7—C20	139.31 (19)	C23—C22—C27—O10	−179.3 (4)
O4—Dy—O7—C20	−124.29 (19)	O9—C22—C27—C26	179.7 (3)
O8—Dy—O7—C20	−0.23 (17)	C23—C22—C27—C26	0.9 (6)
N1—Dy—O7—C20	−157.3 (2)	C28—O10—C27—C22	173.1 (4)
N2—Dy—O7—C20	135.8 (2)	C28—O10—C27—C26	−7.1 (6)
C10—Dy—O7—C20	−171.0 (2)	C25—C26—C27—C22	0.0 (6)
Dy^i^—Dy—O7—C20	−6.31 (18)	C25—C26—C27—O10	−179.8 (4)
C8—O2—C7—C6	−9.5 (6)	C26—C25—C29—C30	−131.6 (3)
C8—O2—C7—C2	172.6 (4)	C24—C25—C29—C30	49.3 (4)
C5—C6—C7—O2	−177.7 (3)	Dy—O11—C30—O12	−24.2 (5)
C5—C6—C7—C2	0.1 (5)	Dy—O11—C30—C29	154.3 (2)
C3—C2—C7—O2	179.2 (3)	Dy^i^—O12—C30—O11	10.4 (6)
O1—C2—C7—O2	−0.1 (5)	Dy^i^—O12—C30—C29	−168.1 (2)
C3—C2—C7—C6	1.2 (6)	C25—C29—C30—O11	−104.8 (3)
O1—C2—C7—C6	−178.1 (3)	C25—C29—C30—O12	73.9 (4)
O8^i^—Dy—O8—C20	169.6 (2)	C42—N1—C31—C32	0.4 (5)
O12^i^—Dy—O8—C20	−110.19 (18)	Dy—N1—C31—C32	−172.8 (3)
O11—Dy—O8—C20	89.53 (18)	N1—C31—C32—C33	1.6 (6)
O3—Dy—O8—C20	−131.90 (19)	C31—C32—C33—C34	−1.7 (6)
O4—Dy—O8—C20	128.75 (17)	C32—C33—C34—C42	0.1 (6)
O7—Dy—O8—C20	0.23 (16)	C32—C33—C34—C35	178.2 (4)
N1—Dy—O8—C20	27.6 (2)	C33—C34—C35—C36	177.8 (4)
N2—Dy—O8—C20	−41.22 (18)	C42—C34—C35—C36	−4.1 (6)
C10—Dy—O8—C20	170.3 (2)	C34—C35—C36—C37	−0.1 (7)
Dy^i^—Dy—O8—C20	169.6 (2)	C35—C36—C37—C38	−177.8 (4)
O8^i^—Dy—O8—Dy^i^	0.0	C35—C36—C37—C41	3.4 (6)
O12^i^—Dy—O8—Dy^i^	80.24 (9)	C41—C37—C38—C39	0.7 (5)
O11—Dy—O8—Dy^i^	−80.05 (9)	C36—C37—C38—C39	−178.1 (4)
O3—Dy—O8—Dy^i^	58.52 (16)	C37—C38—C39—C40	0.4 (6)
O4—Dy—O8—Dy^i^	−40.83 (14)	C41—N2—C40—C39	0.9 (5)
O7—Dy—O8—Dy^i^	−169.35 (13)	Dy—N2—C40—C39	169.9 (3)
N1—Dy—O8—Dy^i^	−142.00 (9)	C38—C39—C40—N2	−1.3 (6)
N2—Dy—O8—Dy^i^	149.20 (9)	C40—N2—C41—C37	0.3 (5)
C10—Dy—O8—Dy^i^	0.7 (2)	Dy—N2—C41—C37	−169.4 (2)
C20—Dy—O8—Dy^i^	−169.6 (2)	C40—N2—C41—C42	−179.5 (3)
C6—C5—C9—C10	104.3 (4)	Dy—N2—C41—C42	10.8 (4)
C4—C5—C9—C10	−73.8 (5)	C38—C37—C41—N2	−1.1 (5)
Dy—O4—C10—O3	−1.5 (3)	C36—C37—C41—N2	177.8 (3)
Dy—O4—C10—C9	178.9 (3)	C38—C37—C41—C42	178.7 (3)
Dy—O3—C10—O4	1.5 (3)	C36—C37—C41—C42	−2.4 (5)
Dy—O3—C10—C9	−178.8 (3)	C31—N1—C42—C34	−2.2 (5)
C5—C9—C10—O4	8.8 (5)	Dy—N1—C42—C34	171.0 (2)
C5—C9—C10—O3	−170.9 (3)	C31—N1—C42—C41	176.6 (3)
O8^i^—Dy—C10—O4	−73.80 (18)	Dy—N1—C42—C41	−10.1 (4)
O12^i^—Dy—C10—O4	−147.15 (17)	C33—C34—C42—N1	2.0 (5)
O11—Dy—C10—O4	−0.71 (19)	C35—C34—C42—N1	−176.3 (3)
O3—Dy—C10—O4	−178.5 (3)	C33—C34—C42—C41	−176.8 (3)
O7—Dy—C10—O4	88.3 (3)	C35—C34—C42—C41	4.9 (5)
O8—Dy—C10—O4	−74.5 (3)	N2—C41—C42—N1	−0.8 (4)
N1—Dy—C10—O4	74.55 (18)	C37—C41—C42—N1	179.4 (3)
N2—Dy—C10—O4	135.87 (18)	N2—C41—C42—C34	178.1 (3)
Dy^i^—Dy—C10—O4	−74.02 (19)	C37—C41—C42—C34	−1.7 (5)

Symmetry codes: (i) −*x*, −*y*+1, −*z*+1.

### Hydrogen-bond geometry (Å, °)

**Table:**

*D*—H···*A*	*D*—H	H···*A*	*D*···*A*	*D*—H···*A*
C1—H1A···O6^ii^	0.96	2.54	3.320 (5)	138
C16—H16A···O3^iii^	0.93	2.53	3.425 (4)	161
C18—H18B···O3^iii^	0.96	2.36	3.262 (4)	156
C21—H21A···O1^iv^	0.96	2.49	3.362 (6)	151
C21—H21A···O2^iv^	0.96	2.43	3.214 (5)	139
C33—H33A···O7^v^	0.93	2.38	3.231 (4)	153
C31—H31A···O4	0.93	2.52	2.977 (4)	111

Symmetry codes: (ii) *x*, *y*+1, *z*−1; (iii) *x*, *y*−1, *z*; (iv) −*x*, −*y*+2, −*z*; (v) −*x*+1, −*y*+1, −*z*+1.
